# Impact of contingent rewards and punishments on employee performance: the interplay of employee engagement

**DOI:** 10.12688/f1000research.144019.2

**Published:** 2024-12-05

**Authors:** Debika Layek, Navin Kumar Koodamara

**Affiliations:** 1 Management, Manipal Academy of Higher Education, Manipal, Karnataka, 576104, India

**Keywords:** Transactional leadership, Contingent rewards, Contingent punishments, Employee Engagement, Employee Performance.

## Abstract

**Background:**

This paper investigated the connection between transactional leadership styles, contingent rewards, punishments, and employee performance while emphasizing employee engagement’s mediating role. Existing research has predominantly focused on isolated associations between contingent rewards, punishment, and employee performance, leaving gaps in the empirical exploration of these mediating mechanisms. To address this research gap, our study has introduced a conceptual framework to understand the multifaceted connection between contingent rewards, punishment, and their effects on employee performance, with a specific emphasis on the mediating function of employee engagement.

**Methods:**

We involved 273 full-time non-clinical healthcare professionals employed in NABH-accredited hospitals in Jharkhand, India. A structured survey instrument was employed for data collection from the specific survey participants, with the investigation of the research hypotheses conducted through the application of partial least squares-structural equation modeling (PLS-SEM).

**Results:**

Preliminary findings suggested that contingent rewards and punishment do not directly influence employee performance. Instead, our study highlighted the critical mediating role of employee engagement, particularly its dimensions of Vigor, absorption, and dedication.

**Conclusions:**

This research has underscored rewards and punishments as essential tools for influencing employee behaviour, motivation, and performance. Employee engagement, as a multifaceted construct, not only benefits individual employees but also significantly impacts overall organizational performance and success.

## Introduction

The rapid evolution of the economic landscape and the dynamic shifts within the business environment have engendered heightened levels of market competition. The rapid growth of India’s healthcare sector highlights the urgent need for effective employee performance measurement. As of 2024, the sector is one of India’s largest employers, with a workforce of 7.5 million people. Due to a significant shortage of healthcare workers, the demand for Indian healthcare professionals is projected to double nationally and globally by 2030 ( Healthcare Industry in India, 2024). This growth, driven by population increase, an aging demographic, rising healthcare costs, and technological advancements, has amplified the demand for high-quality and efficient care. Consequently, measuring employee performance is crucial to addressing these emerging challenges. Considering these circumstances, the cultivation of apt leadership attitudes for navigating an uncertain environment has emerged as a formidable challenge. Consequently, there is an acute necessity for leaders who can not only meet anticipations but also confer a competitive advantage to enhance the performance of employees ( Frangieh & Rusu, 2021; Xie, *et al.*, 2018). Over the past few decades, the realm of leadership has commanded considerable scholarly and practical attention across diverse domains of organizational management. Amongst the numerous leadership paradigms, transformational and transactional leadership have surfaced as the most prominent and widely examined styles within the purview of organizational management ( Baafi, Ansong, Dogbey, & Owusu, 2021; Tyssen, Wald, & Spieth, 2014).

While existing research generally confirms the heightened efficacy of transformational leadership, the present study adopts a more focused approach, centering its inquiry upon transactional leadership. Transactional leaders, in their operational modality, concentrate on the delineation of unambiguous objectives through the interpretation of the reciprocal connection between conferred rewards/punishments and anticipated performance outcomes. Moreover, they consistently engage in the process of furnishing feedback to sustain the concentration of employees on the attainment of their assigned tasks. The influence of transactional leadership on employee performance finds its theoretical underpinning in the tenets of Social Exchange Theory ( Young, Glerum, McCord, & Joseph, 2021). This framework posits that transactional leaders exhibit a profound understanding of the inherent needs and desires of their subordinates, and they proactively articulate how these requisites can be viably fulfilled as proportionate rewards for the enhancement of employee performance. Consequently, leaders operating within this paradigm must judiciously administer a system of rewards and punishments, thereby competently resolving the intricacies associated with the execution of tasks ( Podsakoff, Bommer, Podsakoff, & MacKenzie, 2006).

Drawing from existing research findings, it becomes apparent that while there exists substantiating evidence for the constructive influence of transactional leadership on employee performance, there are also divergent studies that present conflicting perspectives. These contrasting investigations state that transactional leadership can exert a noteworthy adverse impact on employee performance ( Jia, Chen, Mei, & Wu, 2018) or, alternatively, posit that transactional leadership may not have any apparent effect on employee performance ( Baig *et al.*, 2021; Jacobsen, Andersen, Bøllingtoft, & Eriksen, 2022).

In this context, the pivotal roles played by engaged employees are of notable significance. When employees establish a meaningful link with their organization, they tend to exhibit an increased inclination toward enhancing their effectiveness ( Demerouti, Cropanzano, Bakker, & Leiter, 2010). Presently, a substantial body of research literature is progressively corroborating the affirmative influence of Employee Engagement (EE) on Employee Performance (EP) ( Christian, Garza, & Slaughter, 2011; Kanten & Sadullah, 2012). Engaged employees consistently demonstrate a heightened commitment to optimizing their performance and expanding their responsibilities ( Anitha, 2014). This underscores the feasibility of utilizing employee engagement as a predictive factor for employee performance. Notwithstanding the considerable attention accorded to engagement by both practitioners and researchers as a pivotal factor in the realm of work-related dynamics, it is imperative to acknowledge that the definitions and measurement methodologies pertaining to engaged employees, particularly within the context of healthcare services, remain inadequately comprehended. Hence, this research study aims to undertake an exhaustive analysis to elucidate the impact of a transactional leadership style, characterized by contingent rewards and punishments, on employee performance. Furthermore, this study endeavors to illuminate the mediating role of engagement in the context of employee performance.

This study is important to assess the influence of employee engagement on transactional leadership style and performance of employees. This research study represents a novel undertaking, as it constitutes the first effort to assess the variables pertaining to interpersonal relationships within the specific context of India, with a primary emphasis on nonclinical healthcare professionals.

## Literature review

### Transactional leadership

The concept of transactional leadership, as initially formulated by ( Burns & MacGregor, 1978) centers on a dynamic where subordinates willingly engage in a reciprocal relationship with their leaders, anticipating various forms of rewards, whether monetary or non-monetary ( Burns & MacGregor, 1978). This agreed interconnection does not elicit a shift in the roles of the subordinates; rather, it hinges on the transactional leader’s reliance on conventional rewards and punishments to ensure compliance with organizational directives and the attainment of its objectives ( Pathak, Madan, & Srivastava, 2023). This notion, as posited by Bass (1985) posits the existence of an exchange-based association between leaders and their followers. The exchange theory underpinning transactional leadership is pragmatically oriented, emphasizing mutual goal achievement for both parties involved ( Abdelwahed, Soomro, & Shah, 2022). Bass (1985) contends that transactional leaders motivate their followers by establishing clear objectives, outlining direct pathways to goal attainment, providing lucid performance evaluations and feedback, and guaranteeing rewards upon target realization. In this role, these leaders assume the position of evaluators, elucidating the responsibilities of their followers in pursuit of predetermined objectives and administering rewards and punishments based on performance outcomes. Thus, the exchange of interests emerges as a pivotal facet that mutually benefits both leaders and followers ( Politis, 2002).

### Employee engagement

In Blau (1968) the Social Exchange Theory (SET) is explained as a conceptual framework elucidating the dynamics of reciprocity among individuals within various contextual settings. This theoretical construct posits a comprehensive interpretation of the impact of employees’ levels of engagement on their workplace performance ( Saks, 2006). The level of engagement is intricately developed through the lens of exchange theory. Kahn (1990) is credited with pioneering the terminology of ‘employee engagement,’ characterizing it as the extent to which employees manifest tangible involvement, cognitive capability, and emotional attachment to their job responsibilities. Engaged employees are filled with a sense of skill in strategic planning, and decision-making processes. Such motivated individuals exhibit an inclination to channel their utmost efforts and leverage their exceptional abilities to transcend the minimal requisites for organizational advancement ( Breevaart *et al.*, 2014; Kalia & Verma, 2017). In addition to this conceptualization, Schaufeli *et al. *(2002) define engagement as a positive and enriching cognitive state in the occupational context, characterized by three fundamental dimensions. Vigor, the first dimension, manifests through heightened levels of physical and mental energy, coupled with a robust capacity for resilience while engaged in work-related tasks. Dedication, the second dimension, encompasses an emotional landscape characterized by a profound sense of significance, enthusiasm, inspiration, pride, and a constant thirst for challenges associated with one’s professional endeavors. The third dimension, absorption, reflects a state of complete immersion and contented absorption in the execution of one’s occupational responsibilities, signifying a holistic and harmonious alignment between the individual and their work environment ( Schaufeli, Salanova, Roma, & Bakker, 2002).

### Employee performance

Performance is a complex construct encompassing various facets, such as task performance and the contextual roles assumed by employees ( Chaudhary, 2020). It signifies the actions and behaviors exhibited by employees concerning their job-related responsibilities, as well as non-job-related aspects pertinent to the organizational objectives. Furthermore, performance is characterized by the capacity of employees to adapt their conduct in order to meet the requirements of their work ( Park, Lim, Kim, & Kang, 2020). This adaptability contributes to the generation and implementation of innovative and valuable ideas that align with the formal requisites of the job and the proficient fulfillment of assigned duties ( Abdurachman *et al.*, 2023).

### Transactional leadership and employee engagement

Leaders who employ a contingent reward system demonstrate a tendency for establishing explicit objectives and effectively communicating the anticipated outcomes to their followers, thereby eliciting motivation among their subordinates ( Bass & Avolio, 1994). This aligns with the findings of a comprehensive meta-analysis conducted by Judge and Piccolo (2004), which validates the notion that contingent rewards play a pivotal role in enhancing the motivation levels of individuals engaged in the workforce. In contrast, the concept of contingent punishment revolves around an employee’s perception of a leader who meticulously delineates behavioral and performance standards, and subsequently administers various forms of negative feedback, such as criticism or expressions of displeasure, when the employee fails to follow to these established standards ( MacKenzie, Podsakoff, & Rich, Transformational and Transactional Leadership and Salesperson Performance, 2001). This approach underscores the significance of apparent negative reinforcement cues exhibited by leaders, wherein employees face warnings and social disapproval for straying from established regulations and expectations ( Podsakoff, Bommer, Podsakoff, & MacKenzie, 2006). This suggests the extent of an employee’s commitment to their work hinges significantly on the prospect of receiving either rewards or punishments. Consequently, organizations may encounter challenges in fostering change since their leaders are predominantly preoccupied with procedural aspects, tend to focus on identifying faults for punitive measures, and prioritize acknowledging achievements for reward purposes rather than actively cultivating engaged employees ( Thanh & Quang, 2022).

### Employee engagement and employee performances

Considerable attention has been directed towards examining the connection between employee engagement and the evaluation of employee performance. The engagement of employees within the organizational framework yields advantages not only for the employer but also for the employees themselves ( Demerouti, Cropanzano, Bakker, & Leiter, 2010). An establishment of a meaningful connection between employees and the organization invariably promotes a desire for heightened effectiveness among the employees. In contrast, engaged employees consistently outperform their non-engaged counterparts ( Demerouti, Cropanzano, Bakker, & Leiter, 2010). This underscores the intrinsic connection between engagement and employee performance. Studies validate that the initial consequence of employee engagement manifests as augmented employee performance, thereby culminating in amplified organizational performance ( Robbins, Judge, & Vohra, 2019). Engagement offers employees the opportunity to invest themselves in their work and fosters a sense of self-efficacy. Research exploring the ramifications of employee engagement suggests that it can yield positive outcomes in terms of employee well-being and cultivate favourable attitudes towards work and the organization ( Nguyen & Nguyen, 2022). Employee engagement, in essence, denotes the extent to which individuals exhibit identification with their work, actively participate therein, and ascribe importance to their performance as a reflection of their self-worth ( Yandi & Havidz, 2022). A comprehensive synthesis of these findings leads to the inference that work involvement comprises employee participation, marked by a profound dedication to their tasks, transcending personal interests, and emphasizing the overarching importance of the organization. When employees demonstrate a heightened degree of engagement with their employer, their focus extends beyond mere work-related goals, encompassing positive perceptions and emotions, thereby motivating them to exert greater effort in their professional endeavours ( Anitha, 2014). Consequently, a detailed examination of the factors underpinning engagement becomes imperative, as they serve as crucial determinants for predicting employee performance.

### Transactional leadership and employee performance

Transactional leadership can be conceptualized as a form of consensual arrangement between leaders and their subordinates aimed at attaining organizational objectives ( Northouse, 2021). In this leadership paradigm, leaders incentivize employees through the provision of rewards contingent upon the successful execution of tasks that contribute to the maintenance or enhancement of overall employee performance. Transactional leadership model revolves around the fundamental notion of exchanges wherein rewards, both psychological and material, are offered by leaders to their followers in return for their labour ( Donkor & Zhou, 2020). Furthermore, the efficacy of this leadership style is fortified through the implicit or explicit threat of punishments. This theoretical framework operates under the assumption that effective leadership necessitates the cultivation of desired follower behaviours while concurrently mitigating undesirable ones, achieved through the withholding of rewards and the imposition of tangible or psychological penalties ( Alrowwad, Abualoush, & Masa’deh, 2020). In practice, it is typically immediate supervisors who are endowed with the authority to delineate performance objectives for employees, deliver constructive feedback, extend support in exchange for employee efforts, appraise individual performance, and extend recommendations for rewards based on performance evaluations ( Han, Bartol, & Kim, 2015). Leaders who exhibit a propensity for contingent punishment are positively associated with enhanced employee performance ( Podsakoff, Podsakoff, & Kuskova, 2010). They posit that negative feedback, when merited, can exert more constructive effects on employee performance than unwarranted positive feedback. The accountability imposed by leaders upon employees for subpar performance is typically met with greater employee satisfaction concerning their superiors, colleagues, and prospects for career progression ( Podsakoff, Podsakoff, & Kuskova, 2010).

**Hypothesis:**

**H1:** Contingent rewards (CR) have a positive effect on employee performance (EP) of non-clinical healthcare professionals in the healthcare sector.

**H2:** Employee Engagement (vigor, absorption, and dedication) positively mediates the relationship between contingent rewards (CR) and employee performance (EP) of non-clinical healthcare professionals in the healthcare sector.

H2a: CR ➔ Vigor ➔ EP

H2b: CR ➔ Absorption ➔ EP

H2c: CR ➔ Dedication ➔ EP

**H3:** Contingent punishments (CP) have a positive effect on employee performance (EP) of non-clinical healthcare professionals in the healthcare sector.

**H4**: Employee Engagement (vigor, absorption, and dedication) positively mediates the relationship between contingent punishments (CP) and employee performance (EP) of non-clinical healthcare professionals in the healthcare sector.

H4a: CP ➔ Vigor ➔ EP

H4b: CP ➔ Absorption ➔ EP

H4c: CP ➔ Dedication ➔ EP

## Methods

### Ethical statement

This research has been carried out in strict adherence to established ethical guidelines, having received the requisite ethical clearance from the Institutional Ethics Committee at Kasturba Medical College and Kasturba Hospital, Manipal, Karnataka, India as documented under Reference Number IEC1:171/2022. In conducting this non-experimental, survey-based research, we obtained written informed consent from all participants, which was included within the questionnaire document. Participants were granted the option to discontinue their participation in the survey at any juncture, with the assurance that their personal information would be treated with utmost confidentiality.

### Sample and data collection

To examine the hypotheses, we undertook an empirical study with non-clinical healthcare professionals. This study focuses on non-clinical healthcare professionals who play a vital role in supporting patient care alongside clinical staff. These individuals contribute significantly to delivering high-quality healthcare by managing administrative duties, coordinating care activities, overseeing technology, and performing various other essential tasks. By prioritizing the performance of non-clinical healthcare professionals, medical practices can enhance their overall quality of care. The population was composed of all the National Accreditation Board for Hospitals & Healthcare Providers (NABH) -accredited private hospitals located in Jharkhand, India. The data collection process involved obtaining online informed consent from the participants, and an online web-based Microsoft form was prepared and circulated through email. The purpose of the research was explained to the respondents. The present study employed a purposive sampling methodology to gather responses from employees within distinct departments, such as operations, general administration, patient care services, medical insurance, and quality assurance. This approach was chosen due to constraints in the number of available respondents. The primary emphasis of participant selection centered on nonclinical healthcare professionals who are not directly engaged in patient treatment and care services. This targeted sampling strategy was implemented to ensure the acquisition of the most relevant and insightful information for the research.

The final sample consisted of 273 employees. The questionnaire consisted of information on demographic factors, details on the respondent’s gender, age, current organizational designation, total working experience ( Layek & Koodamara, 2023b). The questionnaire consisted of information on demographic factors, details on the respondent’s gender, age, current organizational designation, and total working experience. Data collection took place over a one-month period, commencing on February 10, 2023, and concluding on March 12, 2023. Participants were drawn from five distinct functional departments—operations, general administration, patient care services, medical insurance, and quality assurance. Purposive sampling was adopted for this study as it allows for the intentional selection of participants with specific characteristics relevant to the research focus. By targeting non-clinical healthcare professionals involved in administrative, operational, and support roles, the study ensures that the findings are designed for this group, whose experiences and challenges differ from those of clinical staff. This method enables the collection of focused and relevant insights, enhancing the specificity and relevance of the findings. Exclusion criteria encompassed individuals encountering difficulties comprehending the English language. The dataset ( Layek & Koodamara, 2023a) underwent analysis utilizing Partial Least Squares-Structural Equation Modeling (PLS-SEM), and the entire dataset exhibited an absence of missing values which enhances its integrity, contributing to a thorough and precise portrayal of inter-variable relationships and, consequently, facilitates the explanation of findings derived from the PLS analysis. Data were analysed using PLS-SEM. This statistical tool is endorsed for suitability in contexts characterized by limited sample sizes ( Wong, 2013).

### Measurement scales

We used well-established scales to measure the study variables. Contingent rewards and punishments of transactional leadership style were measured using eight items proposed by Podsakoff *et al. *(1990) and MacKenzie, Podsakoff, and Rich (2001). Employee engagement was measured using the 17 items scale proposed by Schaufeli *et al. *(2002). Employee performance was assessed with 18 items scale proposed by Villagrasaa *et al. *(2019). The respondents assessed all items on a five-point Likert scale (5 = Strongly agree; 4 = Agree, 3 = Neutral, 2 = Disagree, 1 = Strongly disagree) to enhance the functionality and clarity of the questionnaire.

### Common method bias correction

Common method variance bias was effectively assessed Since the data was collected from the respondents in a one-time survey. We used procedural and statistical methods to control for potential common method bias ( Podsakoff, MacKenzie, Podsakoff, & Lee, 2003). Regarding the procedural techniques employed, we took measures to guarantee the confidentiality of the information furnished by the respondents. Furthermore, the study constructs were arranged randomly into the questionnaire to prevent respondents from inferring cause-effect relationships among the constructs. Regarding the statistical procedures, we implemented a full collinearity test based on variance inflation factors (VIFs), following ( Kock & Lynn, 2012). It specifies when a VIF value is greater than 3.3, it indicates collinearity suggesting the existence of common method bias. Our estimations demonstrated that the VIF values fall below the designated threshold, indicating that the issue of common method bias is not of significant concern in this study.

## Results

The engagement of nonclinical healthcare professionals is crucial for several reasons, and it plays a significant role in enhancing their overall performance. When employees are motivated and committed, they are more likely to provide excellent service. The current study reveals the natural inclination of nonclinical healthcare professionals towards roles aligning with their personal preferences, suggesting a propensity for engagement beyond external rewards. These findings emphasize the organizational duty to provide work environments infused with enthusiasm, creating a setting conducive to dynamic and engaged professional experiences.

**Figure 1.**
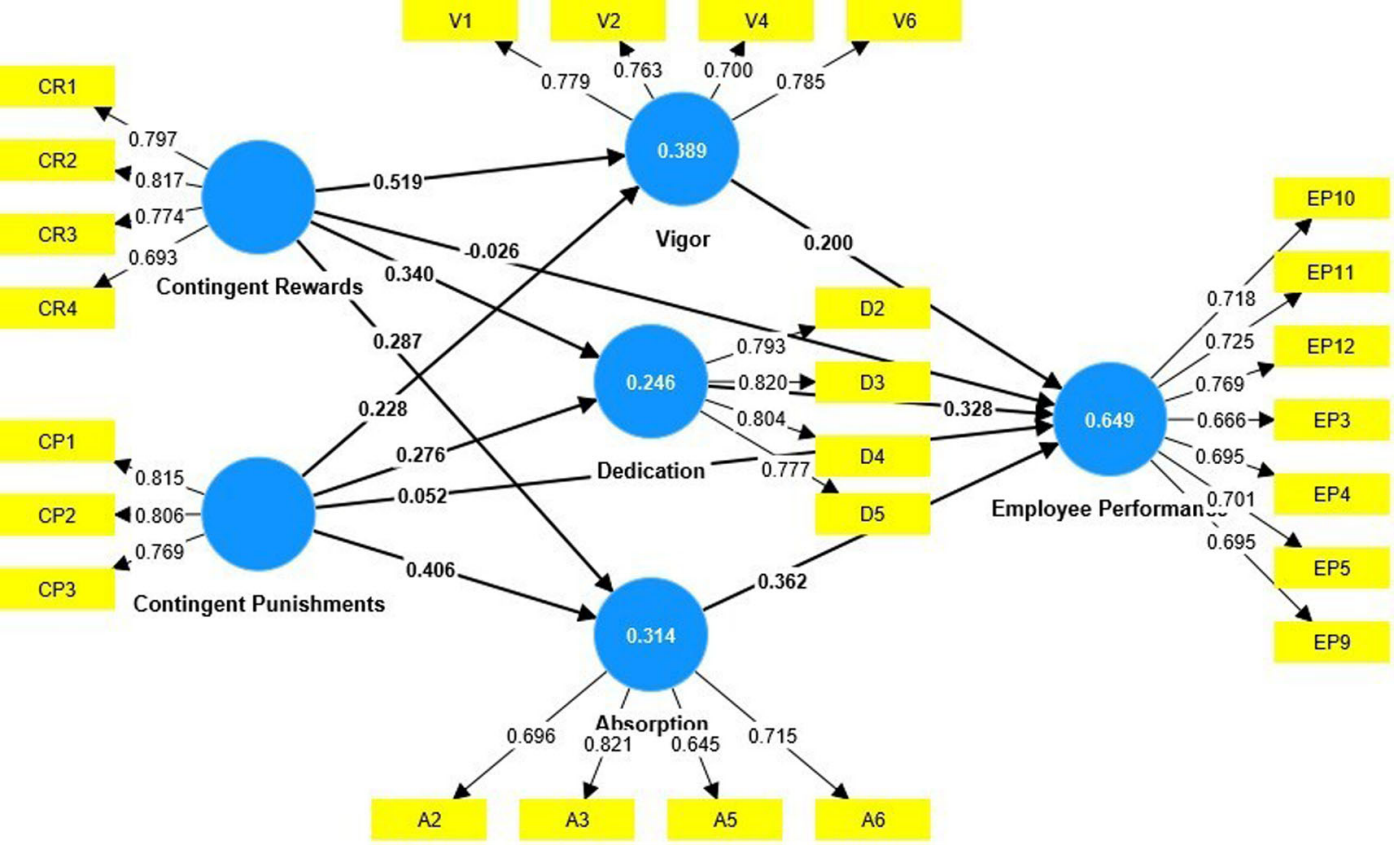
Measurement model.

The research model was tested using partial least squares (PLS). Smart PLS 4 was used ( Figure 1).

### Assessment of measurement model

Table 1 reports the results of the measurement model. To evaluate the adequacy of the measures of the construct model, we assessed the indicator’s individual reliability by examining the loadings of the measures on their corresponding latent constructs. All the construct composite reliability ratios exceeded 0.7, which confirms an adequate correlation between indicators and their respective variables. Convergent validity was assessed which is defined by Henseler, Ringle, & Sinkovics (2009) as a collection of indicators that represent the same mentioned constructs. The latent variables also prove convergent validity as the AVE extracted by the variables is above 0.5 ( Hair Jr. *et al.*, 2021). The discriminant validity of the constructs was assessed and Fornell- Larcker Criterion was employed. Table 1 shows the values for all constructs were less than 0.90, confirming the discriminant validity of the study model. Table 1 indicates that there were no collinearity issues as all the inner VIF (collinearity statistics) values were less than five ( Hair Jr. *et al.*, 2021) and even less than three ( Hair, Risher, Sarstedt, & Ringle, 2019).

**Table 1.** Outer loadings, reliability, VIF values.

Latent variables	Indicators	Loadings	Composite reliability	AVE	VIF	Discriminant validity
Contingent Rewards (CR)	CR1	0.797	0.854	0.595	1.714	0.771
	CR2	0.817			1.784	
	CR3	0.774			1.634	
	CR4	0.693			1.248	
Contingent Punishments (CP)	CP1	0.815	0.839	0.635	1.360	0.797
	CP2	0.806			1.496	
	CP3	0.769			1.375	
Vigor (V)	V1	0.779	0.843	0.574	1.642	0.758
	V2	0.763			1.534	
	V4	0.700			1.260	
	V6	0.785			1.458	
Absorption (A)	A2	0.696	0.812	0.522	1.261	0.722
	A3	0.821			1.565	
	A5	0.645			1.244	
	A6	0.715			1.319	
Dedication (D)	D2	0.793	0.876	0.638	1.650	0.799
	D3	0.820			1.893	
	D4	0.804			1.807	
	D5	0.777			1.522	
Employee Performance (EP)	EP3	0.666	0.877	0.505	1.431	0.710
	EP4	0.695			1.529	
	EP5	0.701			1.520	
	EP9	0.695			1.564	
	EP10	0.718			1.634	
	EP11	0.725			1.636	
	EP12	0.769			1.792	

Source: The Author.

### Assessment of structural model

Furthermore, prior to assessment, the structural model was assessed the bootstrap method was used to evaluate the path coefficients of the structural model. Table 2 shows the t-values, and p-values (95% bias-corrected confidence intervals were evaluated to determine the sign and significance of the path coefficients ( Hair, Risher, Sarstedt, & Ringle, 2019). The value of the structural model offers no support for hypothesis H1 (Contingent Rewards ➔ Employee Performance: β = -0.026, t = 0.529, p = 0.597) and hypothesis H3 (Contingent Punishment ➔ Employee Performance: β = 0.052, t = 1.259, p = 0.208) i.e., there is no significant relationship of the direct effects of contingent rewards and punishments with employee performance.

**Table 2.** Structural model.

Variable relationship	β	t- values	p -values
CR ➔ EP	-0.026	0.529	0.597
CR ➔ EE			
CR ➔ Vigor	0.519	7.456	0.000
CR ➔ Absorption	0.287	3.958	0.000
CR ➔ Dedication	0.340	4.487	0.000
EE ➔ EP			
Vigor ➔ EP	0.200	3.451	0.001
Absorption ➔ EP	0.362	5.896	0.000
Dedication ➔ EP	0.328	5.191	0.000
CP ➔ EE			
CP ➔ Vigor	0.228	3.552	0.000
CP ➔ Absorption	0.406	6.661	0.000
CP ➔ Dedication	0.276	4.569	0.000
CP ➔ EP	0.052	1.259	0.208

Source: The Author.

Note: CR – Contingent Rewards, EP – Employee Performance, EE – Employee Engagement, CP – Contingent Punishment.

### Mediation effect

In order to establish the presence of a mediation effect, three criteria should be met ( Preacher & Hayes, 2008). Firstly, it is important to have a statistically significant association between the independent variable and the mediator. Secondly, the mediator must exhibit a statistically significant relationship with the dependent variable, even after accounting for the influence of the independent variable. Lastly, the indirect effect must attain statistical significance when subjected to rigorous assessment through a bootstrapping procedure.

Table 3 shows the results of the mediation analysis estimations. Aligning with our expectations, all the subdimensions of employee engagement i.e., vigor, absorption, and dedication fully mediate the influence of contingent rewards on employee performance. One of the important conditions for any intervening variable is, that when indirect paths are controlled, the relationship between the predictor variable and outcome variable is no longer significant ( Baron & Kenny, 1986). As shown in Table 3, the direct path between contingent rewards and employee performance is no longer significant (β = -0.026, t = 0.529, p = 0.597). When the path between an independent variable and a dependent variable is reduced to zero, there is a piece of strong evidence for a dominant intervening variable ( Baron & Kenny, 1986). It means the perfect mediating holds when contingent rewards do not affect the employee performance of the employees when their engagement is controlled. Table 3 shows employees’ vigor (V), absorption (A), and dedication (D) as significant mediators in the relationship between contingent rewards and employee performance. In line with these results, hypotheses H2a, H2b, and H2c are accepted.

**Table 3.** Mediation analysis (Contingent Rewards➔ Employee Engagement➔ Employee Performance).

Variable effects	Coefficient	t- values	p-values
Total effects (CR ➔ EP)	0.293	4.332	0.000 *
Direct Effect (CR ➔EP)	-0.026	0.529	0.597
Indirect effects			
CR ➔ V ➔ EP	0.104	3.071	0.002 *
CR ➔ A ➔ EP	0.104	3.393	0.001 *
CR ➔ D ➔ EP	0.111	3.148	0.002 *

Source: The Author.

Note: CR – Contingent Rewards, EP – Employee Performance, EE – Employee Engagement, V – Vigor, A – Absorption, D – Dedication.

* p-value less than 0.05.

Furthermore, Table 4 exhibits the full mediation effect of employee vigor, absorption, and dedication in the relationship between contingent punishments and employee performance, where the direct path between contingent punishments and employee performance is no longer significant (β = 0.052, t = 1.259, p = 0.208). but the total effect between contingent punishments and employee performance is significant (β = 0.335, t = 5.550, p = 0.000). The result leads us to accept hypotheses H4a, H4b, H4c which are supported.

**Table 4.** Mediation analysis (Contingent Punishment ➔ Employee Engagement ➔ Employee Performance).

Variable effects	Coefficient	t-values	p-values
Total effects (CP ➔ EP)	0.335	5.550	0.000 *
Direct Effect (CP ➔EP)	0.052	1.259	0.208
Indirect effects			
CP ➔ V ➔ EP	0.046	2.62	0.009 *
CP ➔ A ➔ EP	0.147	4.456	0.000 *
CP ➔ D ➔ EP	0.091	3.48	0.001 *

Source: The Author.

Note: CP – Contingent Punishment, EP – Employee Performance, EE – Employee Engagement, V – Vigor, A – Absorption, D – Dedication.

* p-value less than 0.05.

## Discussion

This study investigates the underlying mechanisms and conditions that explain why and under what circumstances transactional leadership style specifically the contingent rewards and punishments has impact on employee performance. Specifically, the present study represents one of the first efforts to examine the mediating role of employee engagement of non-clinical healthcare professionals in the relationship between contingent rewards, punishments, and employee performance in the Indian healthcare scenario.

The findings of the study indicate that contingent rewards did not directly forecast employee performance. However, it was observed that all components of employee engagement, namely vigor, absorption, and dedication, showed a full mediation relationship between contingent rewards and employee performance. This observation underscores the significance of employee engagement as a pivotal mechanism linking the impact of contingent rewards to the performance of employees. Notably, individuals exhibit motivation to engage in a task when they possess the confidence that their efforts will yield performance improvements ( Malik, Butt, & Choi, 2015). Empirical evidence further supports the notion that rewards can exert an influence on individual behaviour, contingent upon the perceived value and relevance of these rewards to the recipients ( Schwab, Olian-Gottlieb, & Heneman, 1979). The relationship between rewards and performance has been a subject of debate. It is proposed that the concept of rewards positively predicting employee performance on a situational basis is contingent upon employees possessing high levels of self-engagement and regard. Such attributes are influenced by an intrinsic locus of control, thereby enhancing employee performance ( Malik, Butt, & Choi, 2015). The study underscores the significance of individuals in comprehending leadership phenomena, particularly in the context of rewards being contingent on performance. In this regard, the clarification of task requirements assumes important. In this dynamic, superiors exercise control over rewards, while subordinates employ control over performance. This reciprocal dynamic results in both parties mutually influencing and exerting control over one another ( Yammarino, Spangler, & Dubinsky, 1998).

Furthermore, the study’s results shed light on the impact of contingent punishments which is not directly linked to employees’ performance. Instead, this relationship is indirectly influenced through a full mediation effect involving employee engagement, encompassing factors such as vigor, absorption, and dedication, ultimately affecting employee performance. It is worth noting that punishment, as a managerial practice, possesses popularity and is not easily replaceable by alternative approaches like rewards ( Baron, 2009). However, it is important to recognize that punishment can sometimes have detrimental effects on employee performance ( Podsakoff, Bommer, Podsakoff, & MacKenzie, 2006). The effects of punishment on performance, though, are not straightforward, and the existing literature presents a mixed picture ( Podsakoff, Todor, & Skov, 2017). It is essential for leaders to exercise careful judgment when contemplating the implementation of contingent punishment as a strategy to enhance performance. They must take into account various contextual factors, the nature of the work environment, and the specific individuals involved in the decision-making process to determine the appropriateness and effectiveness of such approach.

In this study, employee engagement assumes the role of a mediating variable, thereby coordinating the underlying mechanism that connects the influence of contingent punishments to employee performance. This finding reaffirms the notion that employees who are deeply engaged in their work are subsequently more inclined to surpass their fundamental job performance ( Scrimpshire, Edwards, Crosby, & Anderson, 2023). The performance of employees is intrinsically linked to their level of engagement, as it is influenced by a multitude of external factors that converge to foster an internal orientation aimed at achieving and delivering high levels of performance. Consequently, the transactional leadership style proves effective in augmenting the performance of non-clinical healthcare professionals, primarily when the influence of contingent rewards and punishments is mediated by employee engagement. This research contributes to the advancement of our comprehension regarding the circumstances under which contingent punishment is associated with job performance. Based on the results, theoretical and managerial implications are discussed.

### Theoretical implications

This research makes several noteworthy contributions to the existing scholarly literature. First and foremost, it responds to the growing demand for a deeper investigation into the various influence mechanisms inherent to transactional leadership ( Zheng, Wu, Xie, & Li, 2019). This study investigates the intricate pathways connecting contingent rewards and punishments with the performance of non-clinical healthcare professionals within the hospital and healthcare sector. Prior research has demonstrated that transactional leadership behaviours are predictive of both task performance and the contextual performance of employees, mediated by constructs such as leader-member exchange ( Kim & Park, 2015), and organizational commitment ( Donkor & Zhou, 2020). Building upon the tenets of social exchange theory, this study extends the existing literature by examining the significance of three distinct sub-dimensions of employee engagement—namely, employee vigor, absorption, and dedication—among healthcare professionals and their consequential impact on performance within the healthcare sector.

Secondly, it is worth noting that there is a limited body of research that has separately probed into the mediating roles of vigor, absorption, and dedication concerning the association between transactional leadership style and employee performance, with a specific emphasis on the contingent rewards and punishment approach ( Deng *et al.*, 2019; Yozgat & Kamanli, 2016). To the best of our knowledge, within the Indian context, there appears to be a dearth of investigations into how two distinct contingent aspects of transactional leadership style function to enhance employee job performance when employee engagement is taken into consideration. Our findings affirm the presence of full mediation effects of employee engagement in augmenting the influence of contingent rewards and punishments on employee performance within the hospital sector.

### Managerial implications

This study presents a range of managerial implications, furnishing professionals in the healthcare industry with a comprehensive framework for comprehending how non-clinical healthcare professionals perceive leadership styles and their consequent influence on job performance.

Firstly, considering our research findings, it is evident that the contingent rewards and punishments approach inherent to transactional leadership does not directly yield improvements in employee performance. Transactional leadership is fundamentally structured around a system of rewards and punishments, and while it can be efficacious in certain contexts, it may not consistently translate into heightened employee performance. In response, managers are advised to consider supplementing transactional leadership with alternative leadership styles that emphasize inspiration and motivation, rather than exclusively relying on the mechanism of rewards and punishments.

Secondly, it is imperative for managers to engage in a critical examination of the performance metrics employed within the context of a transactional leadership model. This assessment should encompass an alignment of these metrics with the organizational objectives, ensuring that they authentically capture the contributions made by employees. The extensive use of transactional leadership, particularly when taken to an excessive degree, can culminate in employee disengagement. Our research reveals a full mediation effect concerning employee engagement, implying that employees may become primarily engaged by external rewards while concurrently experiencing a diminishment in their intrinsic interest in their work or the overarching mission of the organization. Consequently, managers must undertake proactive measures to reignite employee engagement. In cases where employees exhibit dissatisfaction with a transactional leadership style that does not directly foster performance improvement, there is an elevated susceptibility to disengagement among the employees. Addressing this difficulty may necessitate a managerial reassessment of leadership approaches and the overall work environment.

Thirdly, in instances where transactional leadership fails to yield direct improvements in employee performance, it befits managers to exhibit readiness for leadership adaptation. Such adaptation should encompass the exploration of supplementary leadership styles, with a deliberate emphasis on employee engagement. Within the horizon of transactional leadership, rewards are frequently contingent upon specific, tangible outcomes. Consequently, managers may find it advantageous to broaden their range by incorporating diverse modes of recognition and appreciation, aimed at acknowledging and motivating employees, even when the immediate, direct influence on performance remains less obvious.

### Limitations and suggestions for future research

While the present study exhibits robust research design, it is essential to admit certain limitations. Primarily, it is important to recognize that the research employs a cross-sectional design, which, while advantageous in elucidating associations, does not establish causal relationships with certainty. Employing longitudinal research methodologies could provide a more thorough examination of the underlying causal dynamics. Secondly, it is pertinent to note that this study predominantly focuses on analyzing the impact of transactional leadership on the performance of non-clinical healthcare professionals. Future inquiries may opt to delve into the ramifications of transformational leadership on work outcomes or performance within the healthcare domain. This broader perspective would furnish a more comprehensive and comparative understanding of leadership dynamics within healthcare contexts. Despite these acknowledged constraints, this study augments our comprehension of the determinants influencing the performance of non-clinical healthcare professionals, particularly through its emphasis on employee engagement. It underscores the pivotal role played by engaged employees within organizational contexts, thereby offering valuable insights for organizational management and leadership practices.

## Conclusion

This study represents transactional leadership and its constituent components’ impact on the performance of followers, with particular attention directed towards work engagement. The results emphasize the crucial role played by leaders who utilize contingent rewards and punishments in promoting enhanced employee performance, concurrently highlighting the importance of nurturing employee engagement. Our investigation illuminates the inherent inclination of non-clinical healthcare professionals towards roles that resonate with their personal preferences, indicating a tendency towards engagement irrespective of contingent rewards and punishments. These insights underscore the organizational responsibility to offer work experiences infused with enthusiasm, thereby fostering an environment conducive to vibrant and engaged professional lives. It is important to acknowledge that employees serve as the foundational pillar of any organizational entity. Failure to provide them with opportunities for the integration of work and enjoyment within the workplace can engender an escalating sense of disengagement among the employees. Consequently, the cultivation of employee engagement should be perceived not as a single undertaking but as an ongoing, multifaceted process characterized by continual learning, refinement, and proactive measures.

## Data Availability

DRYAD: Contingent reward, punishment and employee performance. https://doi.org/10.5061/dryad.g4f4qrfwk ( Layek & Koodamara, 2023a). The project contains the following underlying data:
• Data file 1.csv (273 Coded respondent’s datasheet). Data are available under the terms of the Creative Commons Zero “No rights reserved” data waiver (CC0 1.0 Public domain dedication). Zenodo: Contingent reward, punishment and employee performance. https://doi.org/10.5281/zenodo.10225424 ( Layek & Koodamara, 2023b). This project contains the following extended data:
•
Blank_Questionnaire_with_likert_scale_(1).docx (Well-established measurement scales for study variables) Data are available under the terms of the Creative Commons Attribution 4.0 International license (CC-BY 4.0).
