# Supplementary material for: Impact of contingent rewards and punishments on employee performance: the interplay of employee engagement
Source: F1000Res. 2024 Dec 5;13:102. Originally published 2024 Feb 16. [Version 2] doi: 10.12688/f1000research.144019.2 (PMC11635189; doi:10.12688/f1000research.144019.2)
Supplement: Peer review history

# Reviewer report: Reviewer response for version 2

**DOI:** 10.5256/f1000research.175194.r346580

**Published:** 2024-12-24

**Authors:** Degdo Suprayitno

**Affiliations:** 1 Institut Ilmu Sosial Dan Manajemen STIAMI, Jakarta, Indonesia

**Is the work clearly and accurately presented and does it cite the current literature?**

 Ensure each discussion section refers to recent research to strengthen the argument.

 If possible, add more citations to recent literature to ensure the study truly reflects the latest developments in the field.

 Overall, the work is clear, and accurate, and cites recent literature well.

**Is the study design appropriate and is the work technically sound?**

 Details on Sample Size Selection:

 A more detailed explanation of how the sample size of 273 was decided (e.g., whether based on statistical calculations or rules of thumb for PLS-SEM) would have improved transparency.

 Context of Method Selection:

 The rationale for selecting only NABH hospitals in Jharkhand could have been explained in more depth to strengthen external validity.

**Are the conclusions drawn adequately supported by the results?**

 Explanation of Direct Path:

 The article mentions that the direct path is not significant but does not provide an in-depth explanation of the implications of the negative coefficient value for contingent rewards (-0.026). Additional explanation would help readers understand the context of this finding.

 Generalization of Results:

 The results are primarily relevant to non-clinical health professionals at NABH hospitals in Jharkhand. The article could highlight this limitation more clearly in the conclusion to avoid overgeneralization.

 Discussion of Practical Effects:

 The article suggests combining other leadership styles with transactional leadership, but this suggestion is not fully supported by research data that focuses only on transactional styles.

Is the work clearly and accurately presented and does it cite the current literature?

Yes

If applicable, is the statistical analysis and its interpretation appropriate?

I cannot comment. A qualified statistician is required.

Are all the source data underlying the results available to ensure full reproducibility?

Yes

Is the study design appropriate and is the work technically sound?

Yes

Are the conclusions drawn adequately supported by the results?

Yes

Are sufficient details of methods and analysis provided to allow replication by others?

Yes

Reviewer Expertise:

Management, Economics, Supply Chain, Logistics and Education.

I confirm that I have read this submission and believe that I have an appropriate level of expertise to confirm that it is of an acceptable scientific standard.

# Reviewer report: Reviewer response for version 2

**DOI:** 10.5256/f1000research.175194.r346157

**Published:** 2024-12-11

**Authors:** Rekha Aranha

**Affiliations:** 1 Christ (Deemed to be University), Bangalore, Karnataka, India

Accepted for indexing

Is the work clearly and accurately presented and does it cite the current literature?

Partly

If applicable, is the statistical analysis and its interpretation appropriate?

Yes

Are all the source data underlying the results available to ensure full reproducibility?

Yes

Is the study design appropriate and is the work technically sound?

Yes

Are the conclusions drawn adequately supported by the results?

Yes

Are sufficient details of methods and analysis provided to allow replication by others?

Yes

Reviewer Expertise:

NA

I confirm that I have read this submission and believe that I have an appropriate level of expertise to confirm that it is of an acceptable scientific standard.

# Reviewer report: Reviewer response for version 2

**DOI:** 10.5256/f1000research.175194.r346158

**Published:** 2024-12-09

**Authors:** Shilpi Saha

**Affiliations:** 1 Manel Srinivas Nayak Institute of Management, Mangaluru, Karnataka, India

This paper is well written. It has mentioned all the theories and literature relevant to the variables.

 The division of findings by the mediating role of vigor, absorption, and dedication in relation to contingent rewards and punishments enhances clarity. The language used is very simple and very clear.

Is the work clearly and accurately presented and does it cite the current literature?

Yes

If applicable, is the statistical analysis and its interpretation appropriate?

Yes

Are all the source data underlying the results available to ensure full reproducibility?

No source data required

Is the study design appropriate and is the work technically sound?

Yes

Are the conclusions drawn adequately supported by the results?

Yes

Are sufficient details of methods and analysis provided to allow replication by others?

Yes

Reviewer Expertise:

Organization Behaviour, Employee engagement, Multiple Organizational commitments, Turnover intention

I confirm that I have read this submission and believe that I have an appropriate level of expertise to confirm that it is of an acceptable scientific standard.

# Reviewer report: Reviewer response for version 1

**DOI:** 10.5256/f1000research.157755.r335650

**Published:** 2024-11-21

**Authors:** Rekha Aranha

**Affiliations:** 1 Christ (Deemed to be University), Bangalore, Karnataka, India

The introduction effectively establishes the broader context of leadership in the modern business environment. There is a clear identification of the research gap and a focus on transactional leadership. The authors have made a good integration of the theoretical framework (Social Exchange Theory) with strong literature support with recent citations and

 clear statement of the study's novelty in the Indian healthcare context.

 The healthcare context is introduced too late. Consider, mentioning it earlier in the introduction explaining why transactional leadership is particularly relevant in healthcare and describing the unique characteristics of nonclinical healthcare professionals

 The sample frame is well-defined with sample frame (NABH-accredited private hospitals in Jharkhand). There is a clear description of the data collection process with established measurement scales

 The theoretical framework can be strengthened by explaining why this theory is particularly relevant to your context, connecting it more explicitly to employee engagement

**Minor Suggestions**

1. Consider adding recent statistics about healthcare sector growth in India to strengthen context
2. Add more specific details about the nonclinical healthcare professional population
3. Consider using subheadings to improve readability
4. Explain why 273 participants are adequate for PLS-SEM
5. Clarify why purposive sampling was chosen over other methods

The article is well-written, the minor changes will enhance the readability

Is the work clearly and accurately presented and does it cite the current literature?

Partly

If applicable, is the statistical analysis and its interpretation appropriate?

Yes

Are all the source data underlying the results available to ensure full reproducibility?

Yes

Is the study design appropriate and is the work technically sound?

Yes

Are the conclusions drawn adequately supported by the results?

Yes

Are sufficient details of methods and analysis provided to allow replication by others?

Yes

Reviewer Expertise:

Organisation culture, STARA, Diversity and inclusivity, employee wellbeing at work

I confirm that I have read this submission and believe that I have an appropriate level of expertise to confirm that it is of an acceptable scientific standard, however I have significant reservations, as outlined above.

# Response

**Published:** 2024-11-22

**Authors:** Navin Koodamara

**Affiliations:** Management, Manipal Academy of Higher Education, Manipal, Karnataka, India

**Title: Impact of contingent rewards and punishments on employee performance: the interplay of employee engagement**

**Reviewer’s Suggestion: 1. Consider adding recent statistics about healthcare sector growth in India to strengthen context**

**Author’s Response: **We appreciate your insightful concern. The following statistical insights information has been added to strengthen the context.

 “The rapid growth of India’s healthcare sector highlights the urgent need for effective employee performance measurement.  As of 2024, the sector is one of India’s largest employers, with a workforce of 7.5 million people. Due to a significant shortage of healthcare workers, the demand for Indian healthcare professionals is projected to double nationally and globally by 2030 (Healthcare Industry in India, 2024). This growth, driven by population increase, an aging demographic, rising healthcare costs, and technological advancements, has amplified the demand for high-quality and efficient care. Consequently, measuring employee performance is crucial to addressing these emerging challenges.”

**Reflected section: **Acknowledging the suggestion, we have highlighted the content mentioned in the 1. **Introduction”** of the manuscript.

**Reviewer’s Suggestion:2. Add more specific details about the nonclinical healthcare professional population.**

**Author’s Response: **The authors have added the following details of the non-clinical healthcare professionals working in the various functional departments of a hospital setting.

  “This study focuses on non-clinical healthcare professionals who play a vital role in supporting patient care alongside clinical staff. These individuals contribute significantly to delivering high-quality healthcare by managing administrative duties, coordinating care activities, overseeing technology, and performing various other essential tasks. By prioritizing the performance of non-clinical healthcare professionals, medical practices can enhance their overall quality of care.”

**Reflected section: **Acknowledging the suggestion, we have highlighted the content mentioned in **“ 3.1. Sample and Data Collection”** of the manuscript.

**Reviewer’s Suggestion:3. Consider using subheadings to improve readability**

**Author’s Response: **Thank you for the insights. Subheadings have been added to the “ **4. Results**” section to produce the findings and improve readability. These are:

 4.1 Assessment of measurement model

 4.2 Assessment of Structural Model

 4.3 Mediation effect.

**Reflected section: **We have highlighted the subheadings mentioned in

**“ 4. Results”** of the manuscript.

**Reviewer’s Suggestion:4. Explain why 273 participants are adequate for PLS-SEM.**

**Author’s Response: **We greatly appreciate your insightful consideration concerning the use of PLS-SEM. This statistical tool is endorsed for its suitability in contexts characterized by limited sample sizes (Wong, 2013). This approach facilitated the examination of hypotheses by estimating path coefficients and T-values.

**Reflected section: **we have highlighted the content mentioned in **“ 3.1. Sample and Data Collection”** of the manuscript.

**Reviewer’s Suggestion:5. Clarify why purposive sampling was chosen over other methods**

**Author’s Response: **We appreciate the concern about the sampling technique adopted for the study. The authors has explained the intention behind choosing purposive sampling  in the following way :

 “Purposive sampling was adopted for this study as it allows for the intentional selection of participants with specific characteristics relevant to the research focus. By targeting non-clinical healthcare professionals involved in administrative, operational, and support roles, the study ensures that the findings are designed for this group, whose experiences and challenges differ from those of clinical staff. This method enables the collection of focused and relevant insights, enhancing the specificity and relevance of the findings.”

**Reflected section: Acknowledging** the suggestion, we have highlighted the content mentioned in **“ 3.1. Sample and Data Collection”** of the manuscript.

# Reviewer report: Reviewer response for version 1

**DOI:** 10.5256/f1000research.157755.r335649

**Published:** 2024-11-08

**Authors:** Shilpi Saha

**Affiliations:** 1 Manel Srinivas Nayak Institute of Management, Mangaluru, Karnataka, India

The article is grounded in well-established theories like Social Exchange Theory, which adds depth to its exploration of transactional leadership's effect on employee performance through employee engagement. 

 The paper clearly addresses a significant gap by focusing on non-clinical healthcare professionals in India, which is a novel and relevant contribution to the field.

 The chosen methodology, a non-experimental, survey-based design with a purposive sampling approach is appropriate for the research question. The study sample of 273 non-clinical healthcare professionals from NABH-accredited hospitals in Jharkhand aligns well with the research aim.

 1. The methodology section is generally well-executed, though it could improve by detailing the selection criteria for participants more explicitly.

 2. Additionally, while the ethical considerations are well-addressed, explaining potential survey biases and how these were managed would enhance transparency.

 Writing style : The article is well-organized, following a logical flow from the introduction and literature review to the discussion and implications. The division of findings by the mediating role of vigor, absorption, and dedication in relation to contingent rewards and punishments enhances clarity.

 1. Some transitions between sections could be smoother, particularly in the results and discussion segments. Adding connecting sentences to emphasize key findings may further aid readability.

 This study represents a strong contribution to understanding transactional leadership in non-clinical healthcare contexts. Minor refinements as suggested above will enhance its rigor and readability.

Is the work clearly and accurately presented and does it cite the current literature?

Yes

If applicable, is the statistical analysis and its interpretation appropriate?

Yes

Are all the source data underlying the results available to ensure full reproducibility?

No source data required

Is the study design appropriate and is the work technically sound?

Yes

Are the conclusions drawn adequately supported by the results?

Yes

Are sufficient details of methods and analysis provided to allow replication by others?

Yes

Reviewer Expertise:

Organization Behaviour, Employee engagement, Multiple Organizational commitments, Turnover intention

I confirm that I have read this submission and believe that I have an appropriate level of expertise to confirm that it is of an acceptable scientific standard.
